# Quercetin negatively regulates IL-1β production in *Pseudomonas aeruginosa*-infected human macrophages through the inhibition of MAPK/NLRP3 inflammasome pathways

**DOI:** 10.1371/journal.pone.0237752

**Published:** 2020-08-20

**Authors:** Kasem Chanjitwiriya, Sittiruk Roytrakul, Duangkamol Kunthalert

**Affiliations:** 1 Department of Microbiology and Parasitology, Faculty of Medical Science, Naresuan University, Phitsanulok, Thailand; 2 National Center for Genetic Engineering and Biotechnology, National Science and Technology Development Agency, Thailand Science Park, Pathumthani, Thailand; 3 Centre of Excellence in Medical Biotechnology, Faculty of Medical Science, Naresuan University, Phitsanulok, Thailand; University of the Pacific, UNITED STATES

## Abstract

*Pseudomonas aeruginosa* remains a leading cause of nosocomial and serious life-threatening infections, and contributes to increased mortality in immunocompromised individuals. *P*. *aeruginosa* infection triggers host immune response and often provokes potent inflammatory mediators, which do not necessarily eradicate the causative pathogen. On the other hand, it causes severe airway damage and eventually decreased lung function. Such unfavorable outcomes of inflammatory injury have necessitated the development of novel effective agents that can combat with *P*. *aeruginosa*-mediated inflammation. Herein, we investigated the potential of quercetin in regulating *P*. *aeruginosa*-induced inflammation, with particular emphasized on the interleukin-1β (IL-1β). Our results showed that quercetin exerted the potent inhibitory activity against the production of IL-1β in macrophages infected by live *P*. *aeruginosa* PAO1, without exhibiting cytotoxicity. According to our settings, such the potent inhibitory activity of quercetin was clearly demonstrated through its ability to efficiently inhibit IL-1β during *P*. *aeruginosa* infection, pre- or even post-infection. In addition, quercetin strongly suppressed MAPK signaling pathway by inhibiting phosphorylation of the p38 MAPK and JNK2, and molecular docking study supported well with this observation. Moreover, quercetin reduced the NLRP3 expression and inhibited the *P*. *aeruginosa*-mediated cleavage of caspase-1 as well as mature IL-1β. These results thus indicated that quercetin inhibition of *P*. *aeruginosa*-induced IL-1β production is mediated by suppressing the initial priming step and by inhibiting the NLRP3 inflammasome activation. Taken together, our findings demonstrated the promising regulatory activity of quercetin against IL-1β production in *P*. *aeruginosa*-infected macrophages, and indicated that quercetin has the potential to be effective as a novel therapeutic agent for treatment of *P*. *aeruginosa*-induced inflammation.

## Introduction

*Pseudomonas aeruginosa* is a Gram-negative bacterium and an opportunistic pathogen capable of infecting humans with compromised host defense mechanisms [1] and causing serious infections and even death [2–4]. Of particular concern are chronic lung infections of patients with cystic fibrosis (CF), a genetic disease that increases susceptibility to respiratory infection [5, 6]. *P*. *aeruginosa* is armed with multiple virulent factors and its infection often induces vigorous inflammatory mediators. When in excess or inappropriate production, these inflammatory mediators are harmful to the host, causing severe airway damage and decreased lung function [7]. Antibiotic treatment of such chronic *P*. *aeruginosa* infection may temporarily suppress the symptoms but they do not necessarily eradicate the causative pathogen. Chronic lung infections with *P*. *aeruginosa* and its associated inflammation is considered to be responsible for significant morbidity and mortality in CF patients [8, 9].

Interleukin-1β (IL-1β) is an important inflammatory mediator of host response to microbial infections, of which activated monocytes and macrophages are the predominant cellular source. A fine-tune control of IL-1β production and secretion is achieved by a two-step process acting both at transcriptional and post-translational levels [10–13]. Initially, the inactive 31-kDa precursor (pro-IL-1β) is synthesized, and subsequently the active/mature form of IL-1β (17-kDa) is generated upon proteolytic cleavage of pro-IL-1β by caspase-1. The first step requires the activation of specific complex pathways, such as mitogen-activated protein kinases (MAPK) and nuclear factor-κB (NF-κB), while the second step requires the activation of inflammasome, including the activation of the proteolytic enzyme caspase-1. The inflammasome is a macromolecular protein complex consisting of at least three components: a NOD-like receptor (NLR) protein such as NLRP3, apoptosis-associated speck-like protein containing a caspase recruitment domain (ASC), and pro-caspase-1 [14, 15]. Accumulating evidence has demonstrated a significant role of IL-1β in *P*. *aeruginosa* infection, potentially in the pathogenesis of CF inflammatory lung disease [16]. Increased IL-1β level was evident in bronchoalveolar lavage fluid from CF patients with infection [17–19] and this increase has been temporally associated with a clinical response to treatment [17]. Human polymorphisms observed in the *IL1B* gene have also been associated with varying degrees of disease severity in CF patients [20]. A murine model based on mice carrying the most common CF mutation F508del CFTR (Cystic Fibrosis Transmembrane Conductance Regulator) also showed that excessive activation of IL-1β correlated with increased bacterial load, inflammation and lung damage [19]. Furthermore, previous different studies have also documented that the reduction of the inflammatory response, especially a decrease in IL-1β, leads to a better outcome in lung infection with *P*. *aeruginosa*. This includes a higher survival rate, reduced damage to the lung tissue and, in particular, a better clearance of the airways and the tissue of the lungs from this bacterium [16]. Although inhaled steroids are usually prescribed as the first-line therapies for chronic inflammatory disease, IL-1β poorly responds to this class of anti-inflammatory drugs [21, 22]. Overall, data available so far point at the cytokine IL-1β as a key target for the development of novel anti-inflammatory drugs for treatment of *P*. *aeruginosa*-mediated chronic disease. Therefore, reduction of such a critical mediator would provide a promising strategy to limit the pathological consequences of *P*. *aeruginosa* infection.

Quercetin, a polyphenol belonging to the class of flavonoids, is widely distributed in fruits, vegetables, and nuts. This natural compound has a wide range of biological actions, including anti-carcinogenic, anti-oxidant and antiviral activities as well as attenuating lipid peroxidation, platelet aggregation and capillary permeability [23–25]. Quercetin has been well-documented for its potent anti-inflammatory activity; this compound showed the inhibition of pro-inflammatory cytokines in various experimental models [26–28]. Previous studies have also demonstrated that quercetin can inhibit NOD-like receptor pyrin domain containing-3 (NLRP3) inflammasome induced by a wide variety of stress signals, including infectious agents [29] and cellular disorders [30–32]. In addition, quercetin has been shown to suppress NLRP3 inflammasome activation in a mouse vasculitis model [33] and a rat model of spinal cord injury [34] as well as ameliorate kidney injury in streptozotocin-induced rats [35]. However, to the best of our knowledge, its immune regulatory function upon pathogenic *P*. *aeruginosa* infection has not yet been reported. The present study therefore accessed the inhibitory activity of quercetin in *P*. *aeruginosa*-infected macrophages, with particular emphasized on the production of a key inflammatory mediator, IL-1β. The possible mechanism involved was also investigated.

## Materials and methods

### Chemicals and antibodies

Quercetin (HPLC purity ≥ 95%), phorbol 12-myristate 13-acetate (PMA), dimethyl sulfoxide (DMSO; ≥ 99.5%) and 3-(4,5-dimethylthiazol-2-yl)-2,5-diphenyltetrazolium bromide (MTT) were purchased from Sigma-Aldrich (St. Louis, MO, USA). Antibodies against IL-1β, NLRP3 (D4D8T), p44/42 MAPK (Erk1/2)(137F5), phospho-p44/42 MAPK (Erk1/2)(Thr202/Tyr204), SAPK/JNK, phospho-SAPK/JNK (Thr183/Tyr185), p38 MAPK and phospho-p38 MAP Kinase (Thr180/Tyr182) were from Cell Signaling Technology (USA). Antibody against caspase-1 was purchased from Santa Cruz Biotechnology (USA) and β-actin antibody was from Abcam (USA). Peroxidase-conjugated AffiniPure goat anti-mouse IgG and anti-rabbit IgG, HRP-linked antibody as secondary antibodies were from Jackson ImmunoResearch Laboratories (USA) and Cell Signaling Technology, respectively.

### Bacterial strain and growth condition

*Pseudomonas aeruginosa* PAO1 was received from the Spanish Type Culture Collection (CECT, Valencia, Spain). This bacterial strain was frozen and kept at ‒ 80°C. Prior to each experiment, two subcultures were prepared on Luria-Bertani (LB) agar (BD Difco™, Le Pont de Claix, France) and incubated under aerobic condition at 37 ^O^C for 24 h. A single colony was then taken and bacterial suspensions were freshly prepared in LB broth or RPMI for subsequent experiments.

### Growth assay

Effect of quercetin on growth of *P*. *aeruginosa* PAO1 were carried out as described previously [36] with some modifications. Bacterial suspension (5×10^5^ CFU/mL) and quercetin at the concentrations from 20–100 μM were incubated at 37 ^O^C for 24 h. Bacterial culture without the test compound was used as the untreated control. After 24 h, bacterial growth was measured at OD600 nm using a microplate reader (BioTek Synergy HT, USA).

### Cell culture and THP-1 differentiation

Human monocytic THP-1 cells (TIB-202) was obtained from American Type Culture Collection (ATCC; Manassas, VA, USA). Cells were cultured in RPMI 1640 medium (HyClone™, Utah, USA) containing 10% (v/v) fetal bovine serum (Gibco, South America), 10 mM 4-(2-hydroxyethyl)-1 piperazineethanesulfonic acid (HEPES; HyClone™), 2 mM L-glutamine (Gibco), 100 U/mL penicillin, 100 μg/mL streptomycin (Gibco) and 0.05 mM 2-mercaptoethanol (Bio-Rad) at 37˚C in a humidified atmosphere of 5% CO_2_. THP-1 cells (1×10^5^ cells/well in 96-well tissue culture plates (Nunc™, Roskilde, Denmark) or 2×10^6^ cells/well in 6-well plates (Nunc™)) were differentiated into macrophages by incubating with 50 ng/mL PMA for 24 h at 37 ^O^C in 5% CO_2_. After washing to remove non-adherent cells, the adherent macrophages were incubated in antibiotic- and FBS-free RPMI for 1 h at 37 ^O^C in 5% CO_2_ and used in subsequent experiments.

### Cell viability assay

Cell viability was evaluated by the MTT assay according to the method previously described [37]. Briefly, differentiated THP-1 cells at a density of 1×10^5^ cells/well were treated with quercetin at the concentrations ranged from 20 to 100 μM, or vehicle (DMSO). Cells without quercetin served as an untreated control. After 24 h, 20 μL MTT solution (5 mg/mL) was added to each well and incubated further for 3 h. Supernatant was removed and 100 μL of DMSO was added to dissolve the formazan crystals. The OD was measured at 540 nm using a microplate reader (Rayto RT-2100C, China). Percentage of cell viability was calculated according to the equation: (OD of treated cells/ OD of untreated cells) × 100.

### Measurement of IL-1β production

Differentiated THP-1 cells (1×10^5^ cells/well) were incubated with *P*. *aeruginosa* PAO1 at the multiplicity of infection (MOI) of 10 in the presence or absence of quercetin (20–100 μM) for 4 h at 37°C with 5% CO_2_, and the supernatant obtained by centrifugation at 4,500 rpm for 15 min at 4°C. In separate experiments, differentiated THP-1 cells were treated with quercetin (20–100 μM) for 2 h prior to *P*. *aeruginosa* exposure, or 2 h after *P*. *aeruginosa* exposure, and culture supernatant collected after 4 h-incubation at 37°C with 5% CO_2._ The levels of IL-1β in the culture supernatant were determined by sandwich enzyme-linked immunosorbent assay (ELISA) using ELISA MAX™ Deluxe Set (BioLegend, San Diego, CA, USA), according to the manufacturer’s instructions.

### Western blot analysis

Differentiated THP-1 cells (2×10^6^ cells/well) were incubated with *P*. *aeruginosa* PAO1 at the MOI of 10 in the presence or absence of quercetin (40–100 μM) for 30 min (for MAPKs) or 4 h (for caspase-1, IL-1β and NLRP3) at 37°C in 5% CO_2_. Supernatants were collected and cell pellets were washed with cold PBS pH 7.4 and resuspended in Pierce^TM^ RIPA lysis buffer (Thermo Scientific, Rockford, IL, USA) containing 1× Halt^TM^ protease and phosphatase inhibitor cocktails (Thermo Scientific). Protein concentration was measured by Bradford protein assay kit (Bio-Rad, USA). Supernatants were precipitated by adding an equal volume of methanol and 1:4 volume of chloroform followed by 10 min centrifugation at 20,000 g at 4°C. After the upper phase was removed, two-volumes of methanol was added and the samples were centrifuged again. The liquid phase was discarded and the pellet dried at 55 ^o^C for 5–10 min. Subsequently, proteins extracted from cell lysates (25 μg) and cell supernatants were dissolved with Laemmli sample buffer (Bio-Rad), heated at 95 ^o^C for 5 min, and then subjected to SDS-PAGE using 10% TGX^TM^ FastCast^TM^ Acrylamide gel (Bio-Rad). Resolved proteins were then transferred to nitrocellulose membranes (Bio-Rad) with semi-dry transfer system (Bio-Rad). The membranes were blocked with 5% skim milk in TTBS (20 mM Tris, 150 mM NaCl, 0.1% Tween20) at room temperature for 1 h. After being washed three times with TTBS, the membranes were incubated overnight with respective primary antibodies against p38 MAPK, phospho-p38 MAP Kinase, SAPK/JNK, phospho-SAPK/JNK, p44/42 MAPK (Erk1/2), phospho-p44/42 MAPK (Erk1/2), caspase-1, IL-1β, NLRP3 and β-actin. After washing with TTBS, the membranes were incubated with peroxidase-conjugated AffiniPure goat anti-mouse IgG or anti-rabbit IgG, HRP-linked antibody, as appropriate, for 1 h at room temperature. The target proteins were visualized by Clarity™ Western ECL Substrate (Bio-Rad) and captured using an ImageQuant LAS 4000 Biomolecular Imager (GE Healthcare Life Sciences, UK). The relative band intensities were quantified using ImageJ software and then normalized with reference to the β-actin as an internal control.

### Docking protocol and their validation

The p38α MAPK (PDB ID: 5XYY) and JNK2 (PDB ID: 3E7O) were chosen for docking studies. The X-ray diffraction structure of p38α MAPK (PDB ID: 5XYY) had a resolution of 1.7 A° [38] and a triazol inhibitor was bound to the ATP-binding of the p38 MAPK. The X-ray diffraction structure of JNK2 (PDB ID: 3E7O) had a resolution of 2.14 A° [39] and N-(3-[5-(1H-1, 2, 4-triazol-3-yl)-1H-indazol-3-yl]phenyl)furan-2-carboxamide was bound to the ATP-pocket of the JNK2. The structure of quercetin (CID = 5280343) was retrieved from the PubChem database [40]. The protein-ligand docking analysis of the p38 MAPK and JNK2 with quercetin was operated using the GOLD version 5.7.1 program of CCDC Software [41]. Predicting the binding affinity and rank-ordering ligands in database screens were implemented by a modified and expanded version of the scoring function. The most suitable method of evaluating the accuracy of a docking procedure is to determine how closely the best fitness score pose predicted by the scoring function resembles an experimental binding mode as determined by X-ray crystallography. The genetic algorithm search parameter was activated to simulate protein-ligand docking with 50 trial runs. Docking validation was performed with an obtained RMSD value of 1.880 A° for 5XYY, 0.325 A° for 3E7O (JNK2 chain A), and 0.270 A° for 3E7O (JNK2 chain B) ensuring the precision and reproducibility of the docking process. The 3D structure was visualized and analyzed using the Discovery Studio Visualizer (Accelrys, Inc., San Diego, CA, USA).

### Statistical analysis

Data are presented as mean ± standard error of mean (SEM) of three independent experiments. Difference between test and control was analyzed by two-tailed student’s *t*-test using SPSS version 20 software (SPSS, Chicago, IL, USA). A *p-*Value of less than 0.05 was considered to be statistically significant.

## Results

### Effect of quercetin on growth of *P*. *aeruginosa*

**Fig 1:**
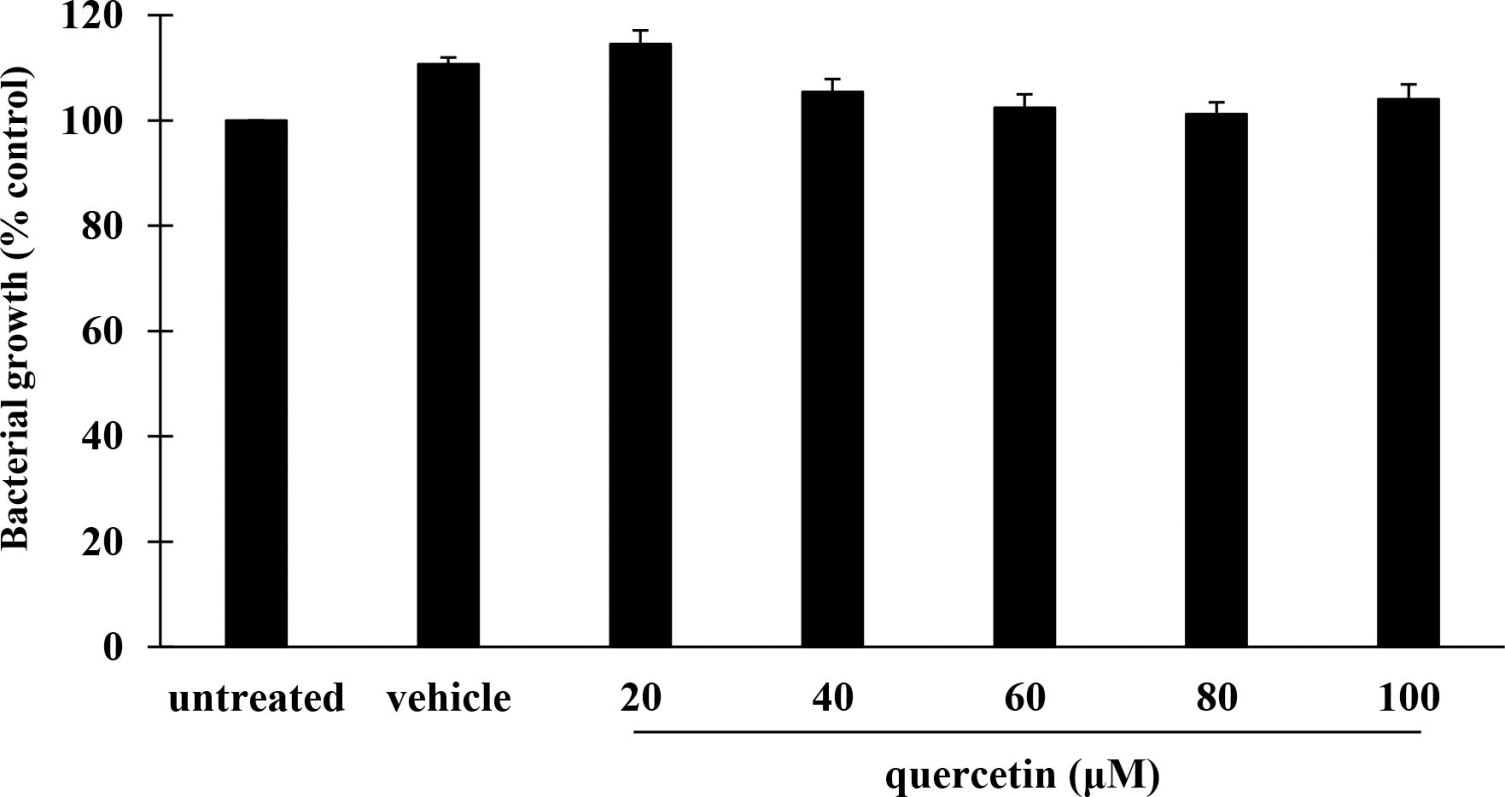
Effect of quercetin on *P*. *aeruginosa* growth. *P*. *aeruginosa* strain PAO1 was treated with quercetin at concentrations ranged from 20–100 μM. After incubation at 37°C for 24 h, bacterial growth was measured at OD600. Values are expressed as mean ± SEM of three independent experiments.

This study used live *P*. *aeruginosa* PAO1 in the infection experiments. We first examined whether quercetin at the concentrations studied had any effect on bacterial viability, and culture-based growth assay was thus performed. As shown in Fig 1, growth of *P*. *aeruginosa* PAO1 after the exposure to quercetin (20–100 μM, final concentration) was comparable to the untreated control. Also, there was no differences in bacterial growth between vehicle and untreated control. These results suggested that quercetin at the concentrations studied had no impact on growth of *P*. *aeruginosa* PAO1, and 20–100 μM of such a compound was used in the following experiments.

### Effect of quercetin on cell viability

**Fig 2:**
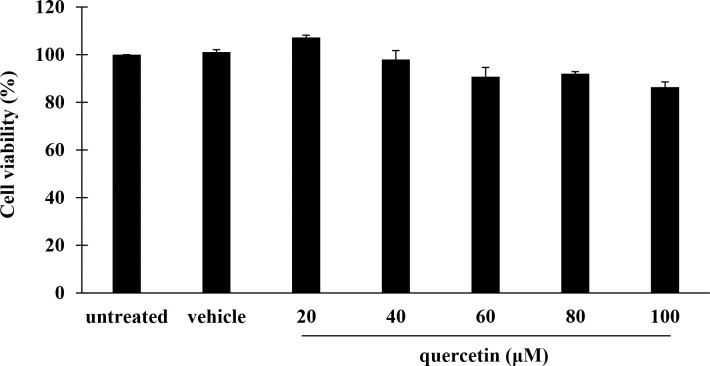
Effect of quercetin on cell viability of THP-1 macrophages. THP-1 macrophages were treated with quercetin at the concentrations ranged from 20–100 μM or vehicle for 48 h and cell viability was assessed by an MTT assay. Values are expressed as mean ± SEM of three independent experiments.

To determine the cytotoxic effect of quercetin against THP-1 macrophages, the MTT assay was conducted. As presented in Fig 2, viability of THP-1 macrophages exposed to quercetin was not significantly changed at any of the concentrations of compound tested, as compared with the untreated control. No significant difference in cell viability between vehicle and untreated controls was observed. These results indicated that quercetin at all concentrations examined, as well as the vehicle was not toxic to THP-1 macrophages.

### Effect of quercetin on IL-1β production in *P*. *aeruginosa*-infected THP-1 macrophages

**Fig 3:**
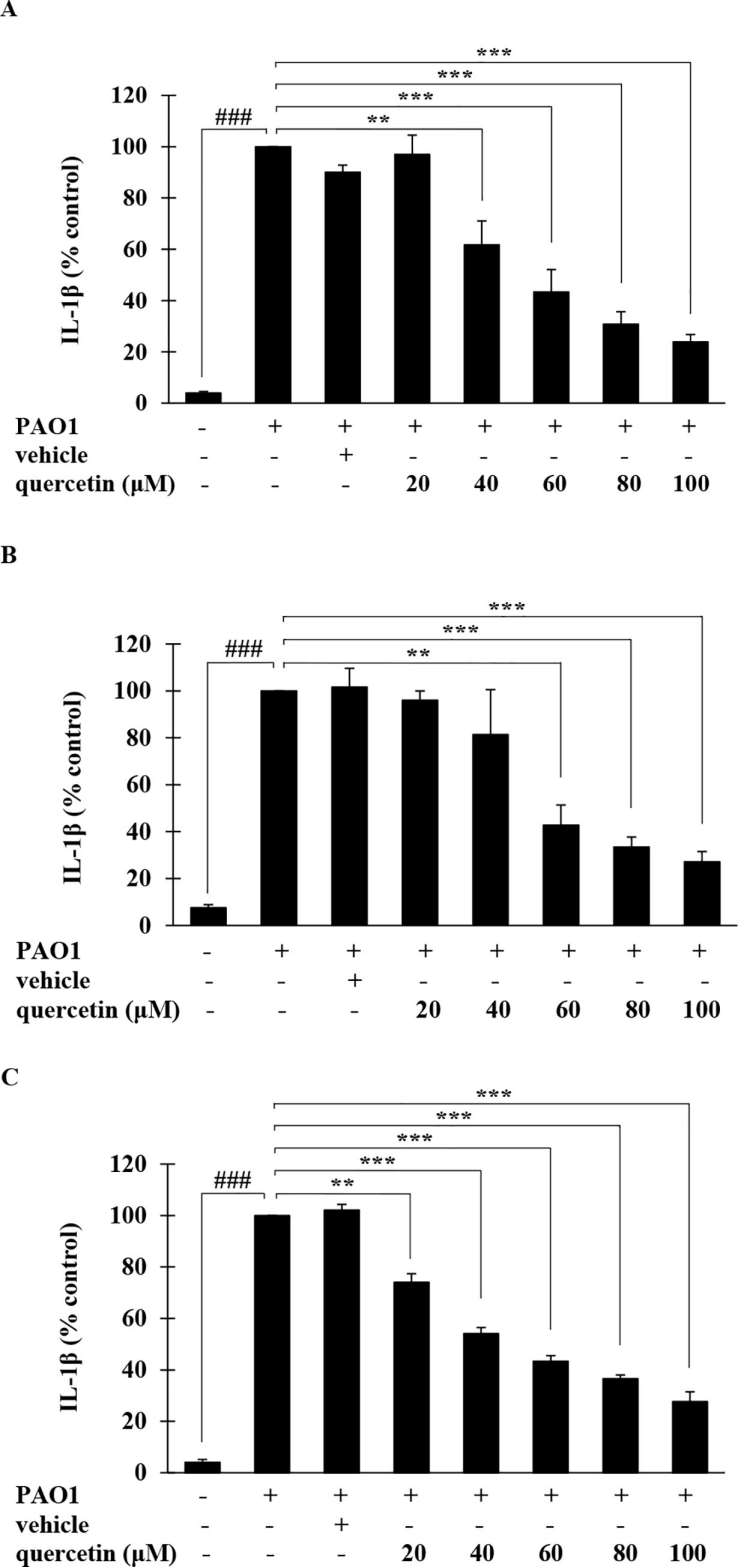
Inhibitory effects of quercetin on IL-1β production in *P*. *aeruginosa*-infected THP-1 macrophages. THP-1 macrophages were treated with quercetin (20–100 μM) at the same time as *P*. *aeruginosa* PAO1 (MOI = 10) for 4 h (A), or 2 h prior to *P*. *aeruginosa* exposure (B), or 2 h after *P*. *aeruginosa* exposure (C), and the levels of IL-1β were measured by sandwich ELISA. Values are represented as mean ± SEM of at least three independent experiments. ### *p* < 0.001 compared with the unstimulated THP-1 macrophages. ** *p* < 0.01 and *** *p* < 0.001 compared with the *P*. *aeruginosa* PAO1-infected THP-1 macrophages.

To evaluate the potential of quercetin in regulating IL-1β produced by *P*. *aeruginosa*-infected THP-1 macrophages, THP-1 cells were exposed to *P*. *aeruginosa* PAO1 simultaneously with quercetin (20–100 μM) for 4 h and the IL-1β level in culture supernatant quantified by ELISA. As shown in Fig 3A, the level of IL-1β was markedly increased in culture supernatant upon infection with *P*. *aeruginosa*, which was significantly and dose-dependently inhibited by quercetin (% inhibition ranged from 2.96–76.11%). To further examine the inhibitory effect of quercetin on *P*. *aeruginosa*-induced production of IL-1β, THP-1 macrophages were treated with quercetin for 2 h prior to *P*. *aeruginosa* exposure, or 2 h after *P*. *aeruginosa* exposure, with each time point also having untreated, vehicle and infection controls. The culture supernatant was collected after 4 h-incubation and the levels of IL-1β assessed. As presented in Fig 3B and 3C, significant reduction in IL-1β level in a dose-related manner was obviously seen after cells were pre- and post-treated with quercetin in relative to *P*. *aeruginosa* exposure, with % inhibition ranged from 4.04–72.87% and 25.94–72.37%, respectively. Particularly, more than 50% inhibition in *P*. *aeruginosa*-induced IL-1β production was evident with quercetin at the concentrations of 60, 80 and 100 μM, and such the inhibitory effects were comparable and not statistically different in all times of quercetin applications. It is of note that only the relatively high doses of quercetin was examined in this study and the inhibition was observed. Since phytochemicals such as allicin, resveratrol and plumbagin have been reported to exhibit biphasic dose-responses; they activate immune cells at low doses and inhibit cell counterparts at high doses [42], careful attention should be made from our results herein.

### Effect of quercetin on the activation of MAPK pathway in *P*. *aeruginosa*-infected THP-1 macrophages

**Fig 4:**
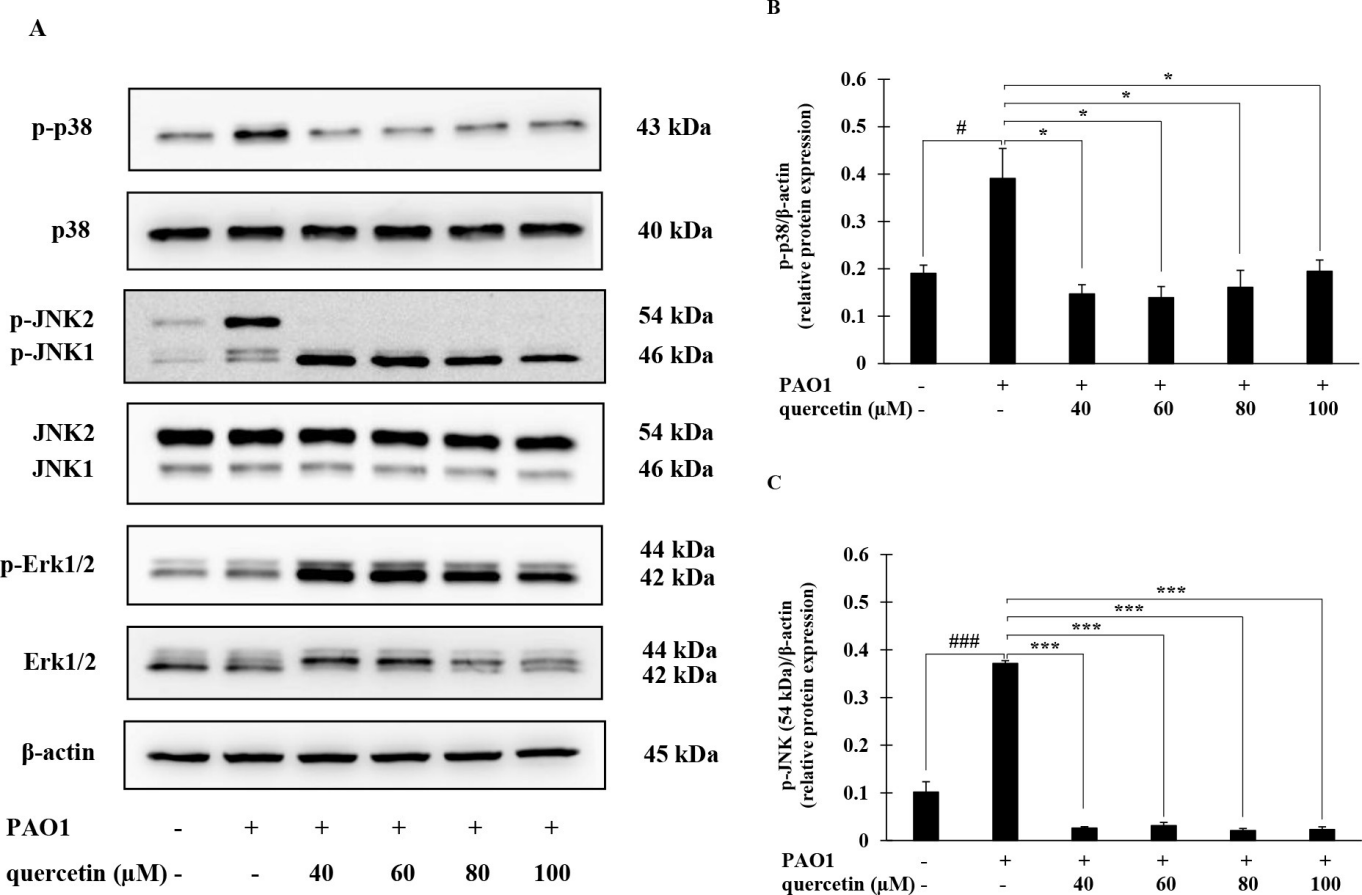
Effects of quercetin on the expression of MAPK pathway-related proteins in THP-1 macrophages infected by *P*. *aeruginosa*. THP-1 macrophages were treated *P*. *aeruginosa* PAO1 at an MOI of 10 in the presence or absence of quercetin (40–100 μM) for 30 min and protein expression was evaluated by Western blotting (A). Bar diagrams showing densitometric analysis of the relative expression of p-p38/β-actin (B) and p-JNK (54 kDa)/β-actin (C), quantified using ImageJ software. Values are represented as mean ± SEM of three independent experiments. # *p* < 0.05 and ### *p* < 0.001 compared with the unstimulated THP-1 macrophages. * *p* < 0.05 and *** *p* < 0.001 compared with the *P*. *aeruginosa* PAO1-infected THP-1 macrophages.

The MAPK signaling pathway has been reported to play an important role in modulating inflammatory response to various exogenous pathogen infection. We further investigated if this pathway may be used by quercetin to exert its inhibitory effect on the release of IL-1β produced by *P*. *aeruginosa*-infected THP-1 macrophages. Changes in the phosphorylation levels of p38, JNK and ERK1/2 in THP-1 macrophages were determined. As shown in Fig 4A, there was an increase in the expression levels of p-p38 and p-JNK, but not p-ERK1/2, in THP-1 macrophages infected by *P*. *aeruginosa* PAO1. However, in the presence of quercetin, the phosphorylation of p38 and JNK (54 kDa) were significantly reduced compared with *P*. *aeruginosa*-treated group without quercetin (Fig 4B and 4C). Moreover, such phosphorylation levels were returned to the baseline seen in the untreated control. No changes in total p38, JNK1/2 and ERK1/2 were observed in all groups.

### Molecular docking

**Fig 5:**
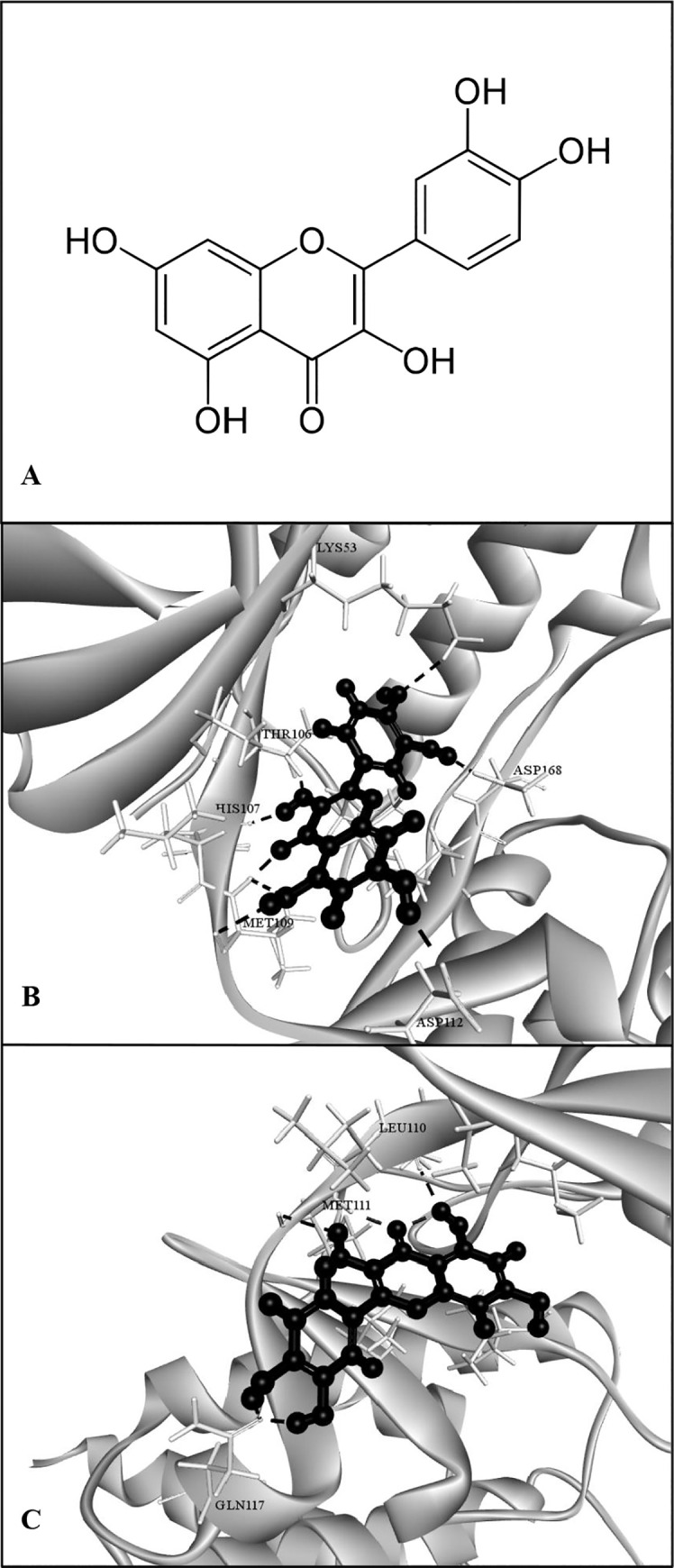
Molecular docking and pose generation. Chemical structure of quercetin is shown (A). A docking study was performed as described in Materials and Methods. Quercetin was docked with p38 MAPK structure (PDB ID: 5XYY) (B), and the JNK2 structure (PDB ID: 3E7O) (C). The proteins are shown in gray cartoon representation, interacting residues are shown in stick representation, the docked quercetin is represented as black ball and sticks, and hydrogen bonds are indicated as broken lines.

To gain some structural insight into the inhibitory mechanism of quercetin, we performed a molecular docking of quercetin *in silico* to the ATP binding pocket of p38 MAPK and JNK using GOLD program [41]. The chemical structure of quercetin, 2-(3,4-dihydroxyphenyl)-3,5,7-trihydroxy-4h-chromen-4-one, is shown in Fig 5A. We found that quercetin formed some favorable connections and docked nicely within the ATP binding sites of p38 MAPK and JNK2 (Fig 5B and 5C). Some important hydrogen bonds were formed between quercetin and both carbonyl backbone and side chains of p38 MAPK including Lys53, Thr106, His107, Met109, Asp112 and Asp168 (Fig 5B). Similarly, quercetin bound to the binding sites of Glu109, Met111 and Gln117, mediated by hydrogen bond interactions in the ATP pocket of the JNK2 structure (Fig 5C).

### Effect of quercetin on the NLRP3 protein expression and IL-1β synthesis in *P*. *aeruginosa*-infected THP-1 macrophages

**Fig 6:**
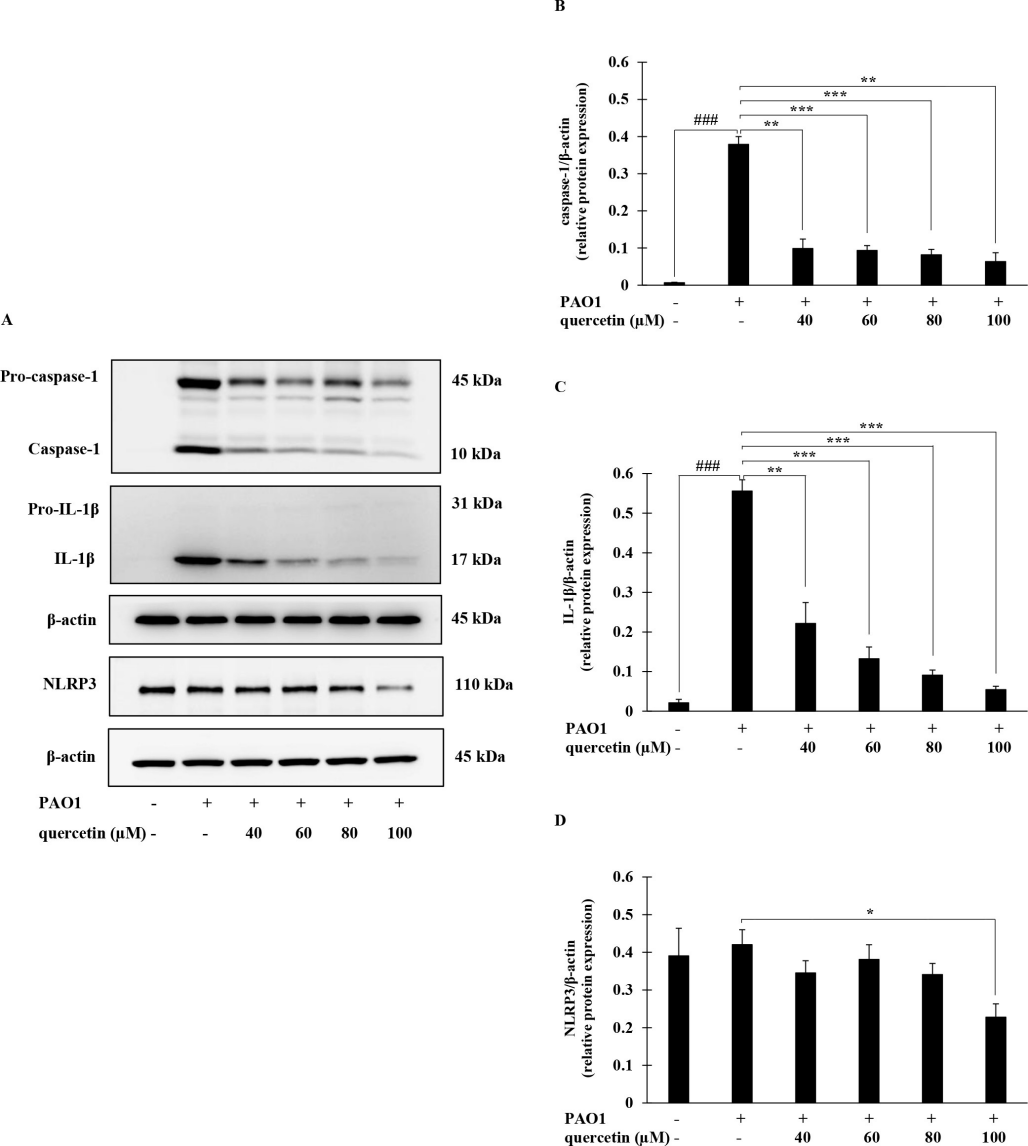
Effects of quercetin on the expression of NLRP3 proteins and IL-1β synthesis in *P*. *aeruginosa*-infected THP-1 macrophages. THP-1 macrophages were treated *P*. *aeruginosa* PAO1 (MOI = 10) in the presence or absence of quercetin (40–100 μM) for 4 hr and protein expression was determined by Western blotting (A). Bar diagrams showing densitometric analysis of the relative expression of caspase-1/β-actin (B), IL-1β/β-actin (C) and NLRP3/β-actin (D), quantified using ImageJ software. Values are represented as mean ± SEM of three independent experiments. ### *p* < 0.001 compared with the unstimulated THP-1 macrophages. * *p* < 0.05, ** *p* < 0.01 and *** *p* < 0.001 compared with the *P*. *aeruginosa* PAO1-infected THP-1 macrophages.

To evaluate the effect of quercetin on the expression of NLRP3 and synthesis of IL-1β in *P*. *aeruginosa*-infected THP-1 macrophages, Western blotting to detect cleaved caspase-1 and IL-1β as well as NLRP3 was performed. As shown in Fig 6, marked increases in the expression of cleaved caspase-1 were observed in THP-1 macrophages infected with *P*. *aeruginosa*, which was significantly decreased in the presence of quercetin. Similarly, substantially increase expression of mature IL-1β was detected in *P*. *aeruginosa*-infected THP-1 macrophages, which was also reduced by quercetin in a concentration-dependent manner (Fig 6C). Additionally, significant reduction in the NLRP3 protein expression was observed by quercetin treatment, in particular at the concentration of 100 μM (Fig 6D).

## Discussion

Despite various impressive pharmacological activities of quercetin, its role in regulating the IL-1β production in *P*. *aeruginosa*-induced inflammation has not been described. This study for the first time reported the potent inhibitory activity of quercetin on the production of IL-1β in macrophages infected by *P*. *aeruginosa*. Herein, live bacteria was used to mimic actual infection and the inhibitory activity of quercetin was accessed through three different strategies. Our results clearly showed that quercetin effectively inhibited the production of IL-1β when applied i) simultaneously, ii) prior to, and iii) after *P*. *aeruginosa* infection (Fig 3), without any significant differences between strategies. Such inhibitory effect was not associated with direct cellular cytotoxicity as MTT assay showed no significant changes in macrophage cell viability upon exposure to quercetin (Fig 2). These results thus indicated the anti-inflammatory potential of quercetin in *P*. *aeruginosa*-induced inflammation. Since IL-1β is a central inflammatory mediator of the responses in *P*. *aeruginosa* infection, our findings therefore suggested that quercetin is a promising therapeutic agent and warrant future development of this compound for the treatment against *P*. *aeruginosa*-induced inflammation.

Unlike other inflammatory cytokines, the production of functional IL-1β is a result of a two-step process which is tightly controlled at the transcriptional and post-translational levels [11–13]. Following initiation of specific complex pathways, such as MAPK and NF-κB, the 31-kDa inactive cytoplasmic IL-1β precursor (pro-IL-1β) is synthesized. Pro-IL-1β is subsequently converted into the bioactive 17-kDa IL-1β by cysteine protease caspase-1. This process requires the assembly of a macromolecular inflammasome complex to which pro-caspase-1 is recruited and cleaved in the presence of a protein belonging to the cytosolic NLR family [14, 15]. During infection, *P*. *aeruginosa* induced the secretion of pro-inflammatory cytokines through the activation of MAPK signaling pathway where the p38 MAPK and JNK are of essential [43, 44]. *P*. *aeruginosa* infection also induces the assembly of the NLRP3 inflammasome and the sequential secretion of caspase-1 and IL-1β in human macrophages [45, 46]. We therefore investigated whether quercetin suppressed such significant pathways involved in IL-1β production induced by *P*. *aeruginosa*. According to our results, quercetin strongly suppressed MAPK signaling pathway by inhibiting phosphorylation of the p38 MAPK and JNK2. Molecular docking study also provided deep insights into inhibitory mechanism of quercetin and this further supported that p38 MAPK and JNK2 are specific targets of quercetin. Not only the components of MAPK pathway, quercetin also suppressed the NLRP3 expression and inhibited the *P*. *aeruginosa*-mediated cleavage of caspase-1 (Fig 6). Consistent with this, mature IL-1β production was decreased following quercetin treatment (Fig 6). These results thus indicated that quercetin inhibition of *P*. *aeruginosa*-induced IL-1β production is mediated by suppressing the initial priming step as well as by inhibiting the inflammasome activation. The fact that effectiveness of inhibition of quercetin was comparable and not statistically different when this compound was added at the same time, prior to or even after *P*. *aeruginosa* infection (Fig 3), it is likely that quercetin inhibition in this setting may act on signaling rather than direct interaction with *P*. *aeruginosa*. Previous studies on flavonoid uptake and transport in various cell lines have described that the transport mechanism of quercetin is through passive diffusion [47]. In this regard, it is possible that quercetin may penetrate macrophages and subsequently interfere with signaling molecules in the signaling pathways involved in the production of functional IL-1β. Since p38 MAPK and JNK2 are recognized for its role regulating the *P*. *aeruginosa* elicited IL-1β [48], and NLRP3 and caspase-1 is essential components of inflammasome, our findings therefore proved the potential of quercetin on the regulation in *P*. *aeruginosa*-induced production of IL-1β.

## Conclusions

This study provided clear evidence that quercetin possessed the potent inhibitory activity against the production of IL-1β in macrophages infected by live *P*. *aeruginosa*, without exhibiting cytotoxicity. Our findings also revealed that quercetin inhibition of *P*. *aeruginosa*-induced IL-1β production is mediated through MAPK (p38 and JNK2) and NLRP3 inflammasome activation pathways. Since effectiveness in inhibition of quercetin was apparently demonstrated, we therefore propose that quercetin may be applicable as a potential prophylactic/therapeutic molecule against *P*. *aeruginosa*-mediated inflammation. Nevertheless, further investigations in an *in vivo P*. *aeruginosa*-infection model are required to support future therapeutic applications of this compound.

## Supporting information

S1 File The primary data underlying our results. (DOCX)

S1 Raw images The images of the entire blots used to generate Fig 4A. (PDF)

S2 Raw images The images of the entire blots used to generate Fig 6A. (PDF)
