# Supplementary material for: Quercetin negatively regulates IL-1β production in Pseudomonas aeruginosa-infected human macrophages through the inhibition of MAPK/NLRP3 inflammasome pathways
Source: PLoS One. 2020 Aug 20;15(8):e0237752. doi: 10.1371/journal.pone.0237752 (PMC7446918; doi:10.1371/journal.pone.0237752)
Supplement: S1 File — (DOCX) [file pone.0237752.s001.docx]

**Fig 1. Effect of quercetin on *P. aeruginosa* growth.**

| **Bacterial growth (% control)** | | | | | | |
| --- | --- | --- | --- | --- | --- | --- |
| **Treatment** | **concentration** | **Exp. 1** | **Exp. 2** | **Exp. 3** | **x̄** | **SEM** |
| **untreated** | - | 100.000 | 100.000 | 100.000 | 100.000 | 0.000 |
| **vehicle** | - | 110.930 | 108.300 | 112.789 | 110.673 | 1.302 |
| **quercetin** | 20 µM | 110.678 | 113.439 | 119.456 | 114.524 | 2.591 |
|  | 40 µM | 103.769 | 102.372 | 110.204 | 105.448 | 2.412 |
|  | 60 µM | 99.874 | 100.000 | 107.483 | 102.452 | 2.516 |
|  | 80 µM | 98.618 | 99.209 | 105.714 | 101.181 | 2.273 |
|  | 100 µM | 107.538 | 98.419 | 106.122 | 104.026 | 2.833 |

**Fig 2. Effect of quercetin on cell viability of THP-1 macrophages.**

| **Cell viability (%)** | | | | | | |
| --- | --- | --- | --- | --- | --- | --- |
| **Treatment** | **concentration** | **Exp. 1** | **Exp. 2** | **Exp. 3** | **x̄** | **SEM** |
| **untreated** | - | 100.000 | 100.000 | 100.000 | 100.000 | 0.000 |
| **vehicle** | - | 101.421 | 99.209 | 102.663 | 101.098 | 1.010 |
| **quercetin** | 20 µM | 105.415 | 108.683 | 107.572 | 107.223 | 0.959 |
|  | 40 µM | 94.304 | 94.140 | 105.483 | 97.976 | 3.754 |
|  | 60 µM | 90.654 | 84.080 | 97.546 | 90.760 | 3.887 |
|  | 80 µM | 92.419 | 90.399 | 93.264 | 92.027 | 0.850 |
|  | 100 µM | 87.204 | 89.693 | 82.350 | 86.416 | 2.156 |

**Fig 3. Inhibitory effects of quercetin on *P. aeruginosa*-induced IL-1β production.**

**Fig 3A. Co-culture**

| **IL-1β (% control)** | | | | | | |
| --- | --- | --- | --- | --- | --- | --- |
| **Treatment** | **Exp. 1** | **Exp. 2** | **Exp. 3** | **Exp. 4** | **x̄** | **SEM** |
| **untreated** | 2.848 | 4.895 | 4.670 | 3.374 | 3.947 | 0.573 |
| **PAO1** | 100.000 | 100.000 | 100.000 | 100.000 | 100.000 | 0.000 |
| **PAO1 + vehicle** | 86.009 | 96.028 | 86.259 | 91.799 | 90.024 | 2.779 |
| **PAO1 + quercetin 20 µM** | 79.861 | 110.090 | 103.183 | 94.878 | 97.003 | 7.512 |
| **PAO1 + quercetin 40 µM** | 45.699 | 55.154 | 83.627 | 62.410 | 61.723 | 9.311 |
| **PAO1 + quercetin 60 µM** | 31.646 | 33.564 | 64.622 | 43.602 | 43.359 | 8.726 |
| **PAO1 + quercetin 80 µM** | 24.305 | 24.851 | 42.618 | 31.107 | 30.720 | 4.914 |
| **PAO1 + quercetin 100 µM** | 18.408 | 21.808 | 29.949 | 25.389 | 23.888 | 2.855 |

**Fig 3B. Pre-treatment**

| **IL-1β (% control)** | | | | | |
| --- | --- | --- | --- | --- | --- |
| **Treatment** | **Exp. 1** | **Exp. 2** | **Exp. 3** | **x̄** | **SEM** |
| **untreated** | 8.836 | 9.586 | 4.287 | 7.570 | 1.282 |
| **PAO1** | 100.000 | 100.000 | 100.000 | 100.000 | 0.000 |
| **PAO1 + vehicle** | 88.743 | 99.749 | 116.374 | 101.622 | 8.031 |
| **PAO1 + quercetin 20 µM** | 100.000 | 100.000 | 87.890 | 95.963 | 4.037 |
| **PAO1 + quercetin 40 µM** | 61.153 | 119.757 | 62.988 | 81.299 | 19.236 |
| **PAO1 + quercetin 60 µM** | 29.418 | 58.853 | 39.877 | 42.719 | 8.615 |
| **PAO1 + quercetin 80 µM** | 24.946 | 36.333 | 38.968 | 33.416 | 4.302 |
| **PAO1 + quercetin 100 µM** | 18.534 | 32.859 | 29.985 | 27.126 | 4.375 |

**Fig 3C. Post-treatment**

| **IL-1β (% control)** | | | | | |
| --- | --- | --- | --- | --- | --- |
| **Treatment** | **Exp. 1** | **Exp. 2** | **Exp. 3** | **x̄** | **SEM** |
| **untreated** | 2.184 | 4.018 | 5.972 | 4.058 | 1.094 |
| **PAO1** | 100.000 | 100.000 | 100.000 | 100.000 | 0.000 |
| **PAO1 + vehicle** | 97.693 | 103.303 | 105.252 | 102.082 | 2.266 |
| **PAO1 + quercetin 20 µM** | 68.176 | 74.215 | 79.763 | 74.051 | 3.346 |
| **PAO1 + quercetin 40 µM** | 53.143 | 58.595 | 50.498 | 54.079 | 2.384 |
| **PAO1 + quercetin 60 µM** | 39.279 | 44.284 | 46.604 | 43.389 | 2.161 |
| **PAO1 + quercetin 80 µM** | 39.458 | 34.546 | 35.648 | 36.551 | 1.488 |
| **PAO1 + quercetin 100 µM** | 24.441 | 23.198 | 35.263 | 27.634 | 3.831 |

**Fig 4. Effects of quercetin on the expression of MAPK pathway-related proteins in THP-1 macrophages infected by *P. aeruginosa*.**

**Fig 4B. p-p38/β-actin (relative protein expression)**

| **p-p38/β-actin** | | | | | |
| --- | --- | --- | --- | --- | --- |
| **Treatment** | **Exp. 1** | **Exp. 2** | **Exp. 3** | **x̄** | **SEM** |
| **untreated** | 0.206 | 0.155 | 0.209 | 0.190 | 0.017 |
| **PAO1** | 0.435 | 0.266 | 0.471 | 0.391 | 0.063 |
| **PAO1 + quercetin 40 µM** | 0.158 | 0.109 | 0.174 | 0.147 | 0.020 |
| **PAO1 + quercetin 60 µM** | 0.152 | 0.093 | 0.172 | 0.139 | 0.024 |
| **PAO1 + quercetin 80 µM** | 0.215 | 0.093 | 0.175 | 0.161 | 0.036 |
| **PAO1 + quercetin 100 µM** | 0.242 | 0.174 | 0.168 | 0.195 | 0.024 |

**Fig 4C. p-JNK (54 kDa)/β-actin (relative protein expression)**

| **p-JNK (54 kDa)/β-actin** | | | | | |
| --- | --- | --- | --- | --- | --- |
| **Treatment** | **Exp. 1** | **Exp. 2** | **Exp. 3** | **x̄** | **SEM** |
| **untreated** | 0.076 | 0.144 | 0.085 | 0.102 | 0.021 |
| **PAO1** | 0.63 | 0.382 | 0.369 | 0.371 | 0.006 |
| **PAO1 + quercetin 40 µM** | 0.021 | 0.031 | 0.025 | 0.026 | 0.003 |
| **PAO1 + quercetin 60 µM** | 0.042 | 0.033 | 0.018 | 0.031 | 0.007 |
| **PAO1 + quercetin 80 µM** | 0.024 | 0.0286 | 0.012 | 0.021 | 0.005 |
| **PAO1 + quercetin 100 µM** | 0.028 | 0.029 | 0.011 | 0.023 | 0.006 |

**Fig 6. Effects of quercetin on the expression of NLRP3 proteins and IL-1β synthesis in THP-1 macrophages infected by *P. aeruginosa*.**

**Fig 6B.**  **caspase-1/β-actin (relative protein expression)**

| **caspase-1/β-actin** | | | | | |
| --- | --- | --- | --- | --- | --- |
| **Treatment** | **Exp. 1** | **Exp. 2** | **Exp. 3** | **x̄** | **SEM** |
| **untreated** | 0.009 | 0.006 | 0.004 | 0.006 | 0.001 |
| **PAO1** | 0.380 | 0.342 | 0.415 | 0.379 | 0.021 |
| **PAO1 + quercetin 40 µM** | 0.149 | 0.075 | 0.072 | 0.099 | 0.025 |
| **PAO1 + quercetin 60 µM** | 0.096 | 0.115 | 0.070 | 0.093 | 0.013 |
| **PAO1 + quercetin 80 µM** | 0.078 | 0.109 | 0.058 | 0.082 | 0.015 |
| **PAO1 + quercetin 100 µM** | 0.035 | 0.111 | 0.044 | 0.063 | 0.024 |

**Fig 6C. IL-1β/β-actin** **(relative protein expression)**

| **IL-1β/β-actin** | | | | | |
| --- | --- | --- | --- | --- | --- |
| **Treatment** | **Exp. 1** | **Exp. 2** | **Exp. 3** | **x̄** | **SEM** |
| **untreated** | 0.017 | 0.009 | 0.038 | 0.021 | 0.008 |
| **PAO1** | 0.538 | 0.611 | 0.518 | 0.556 | 0.028 |
| **PAO1 + quercetin 40 µM** | 0.303 | 0.122 | 0.239 | 0.221 | 0.053 |
| **PAO1 + quercetin 60 µM** | 0.175 | 0.076 | 0.146 | 0.132 | 0.030 |
| **PAO1 + quercetin 80 µM** | 0.113 | 0.068 | 0.092 | 0.091 | 0.013 |
| **PAO1 + quercetin 100 µM** | 0.055 | 0.039 | 0.069 | 0.054 | 0.009 |

**Fig 6D. NLRP3/β-actin (relative protein expression)**

| **NLRP3/β-actin** | | | | | |
| --- | --- | --- | --- | --- | --- |
| **Treatment** | **Exp. 1** | **Exp. 2** | **Exp. 3** | **x̄** | **SEM** |
| **untreated** | 0.361 | 0.200 | 0.463 | 0.539 | 0.391 |
| **PAO1** | 0.340 | 0.481 | 0.364 | 0.496 | 0.420 |
| **PAO1 + quercetin 40 µM** | 0.310 | 0.291 | 0.342 | 0.437 | 0.345 |
| **PAO1 + quercetin 60 µM** | 0.308 | 0.397 | 0.337 | 0.484 | 0.381 |
| **PAO1 + quercetin 80 µM** | 0.264 | 0.350 | 0.343 | 0.407 | 0.341 |
| **PAO1 + quercetin 100 µM** | 0.184 | 0.333 | 0.193 | 0.203 | 0.228 |
