# Supplementary material for: Quercetin negatively regulates IL-1β production in Pseudomonas aeruginosa-infected human macrophages through the inhibition of MAPK/NLRP3 inflammasome pathways
Source: PLoS One. 2020 Aug 20;15(8):e0237752. doi: 10.1371/journal.pone.0237752 (PMC7446918; doi:10.1371/journal.pone.0237752)

Fig 4A

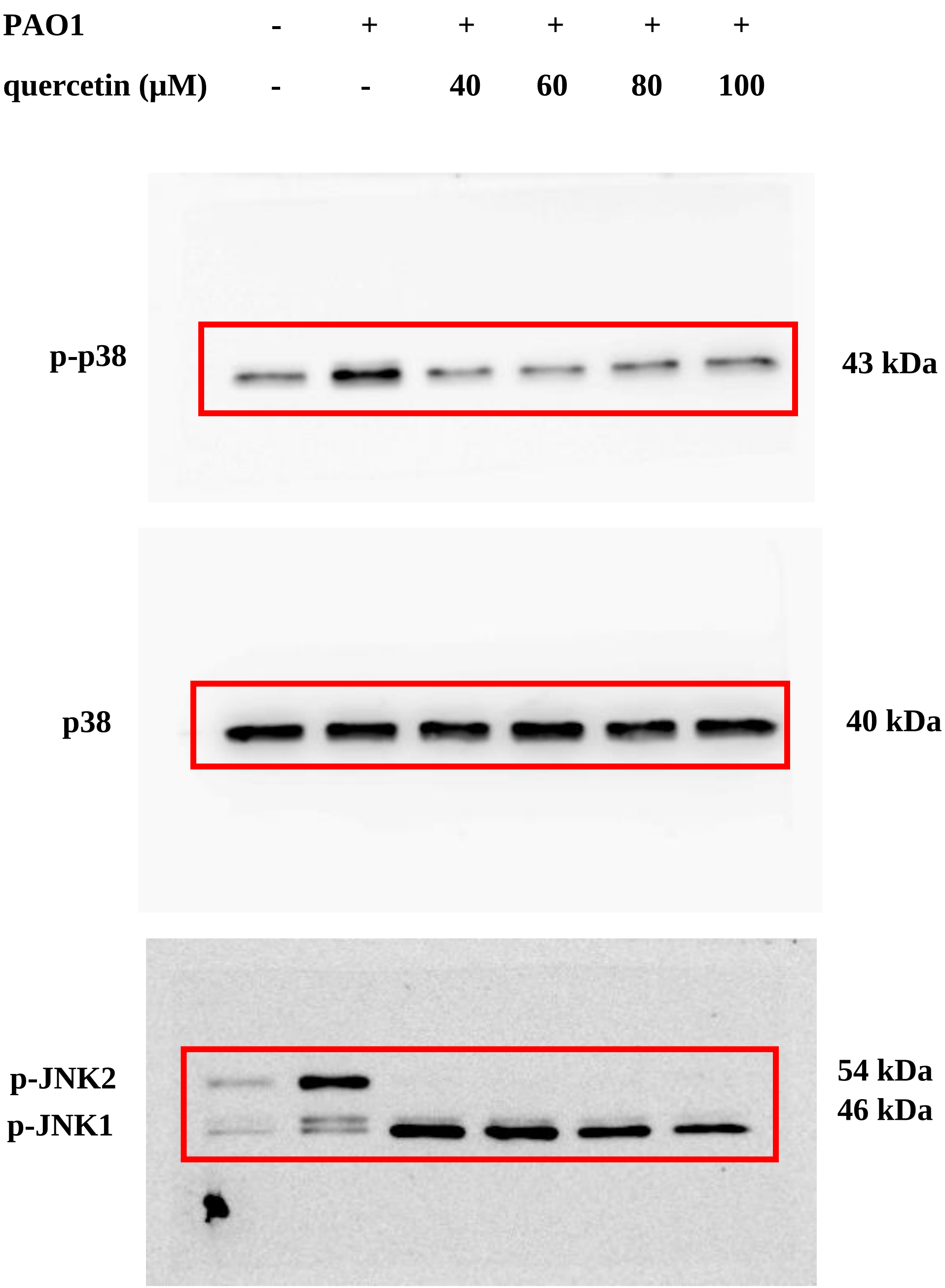

PAO1 - + + + + +
quercetin (μM) - - 40 60 80 100
p-p38
43 kDa
p38
40 kDa
p-JNK2
p-JNK1
54 kDa
46 kDa

Fig 4A

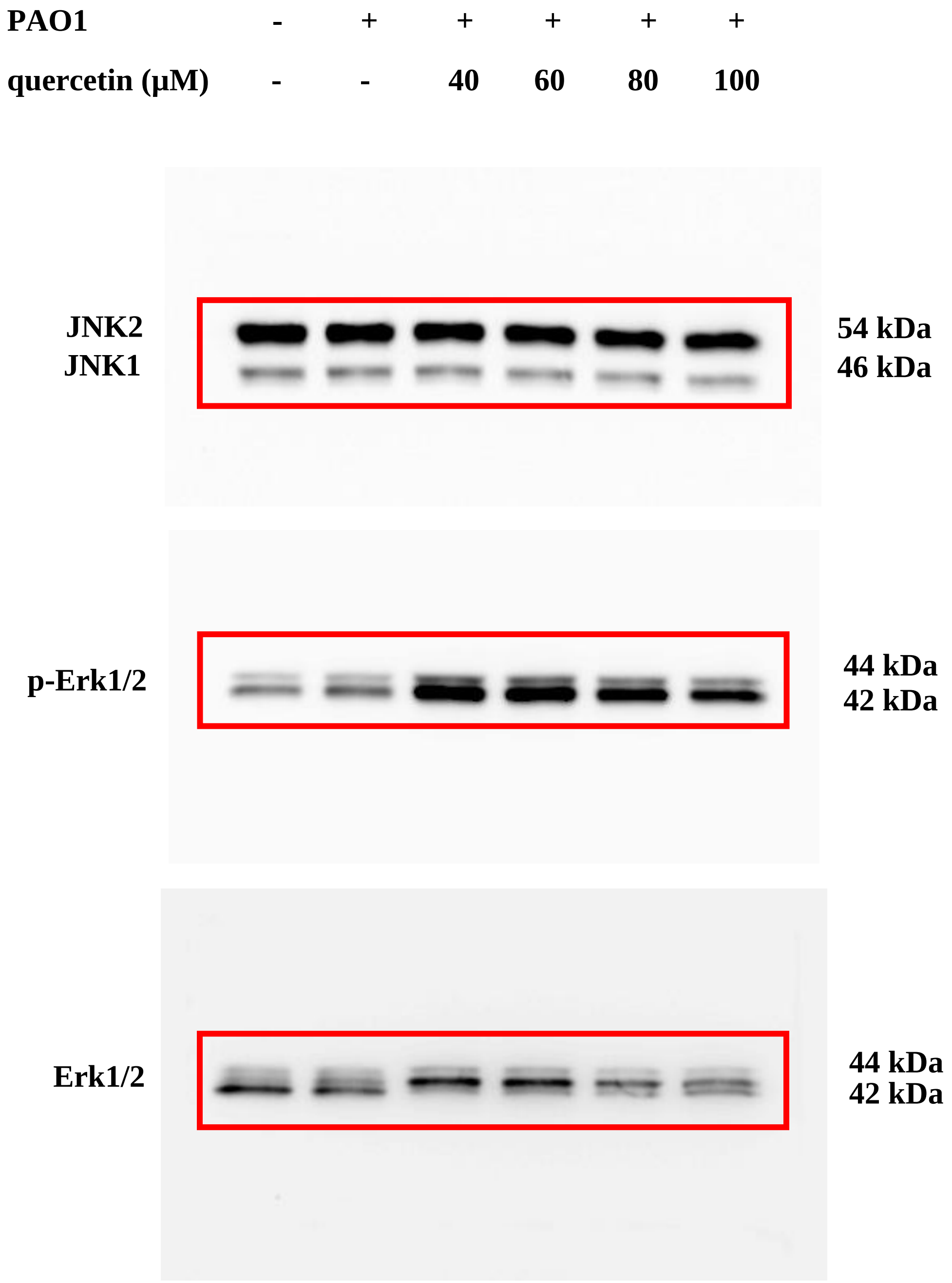
PAO1 - + + + + +
quercetin (μM) - - 40 60 80 100
JNK2
JNK1
54 kDa
46 kDa
p-Erk1/2
44 kDa
42 kDa
Erk1/2
44 kDa
42 kDa

Fig 4A

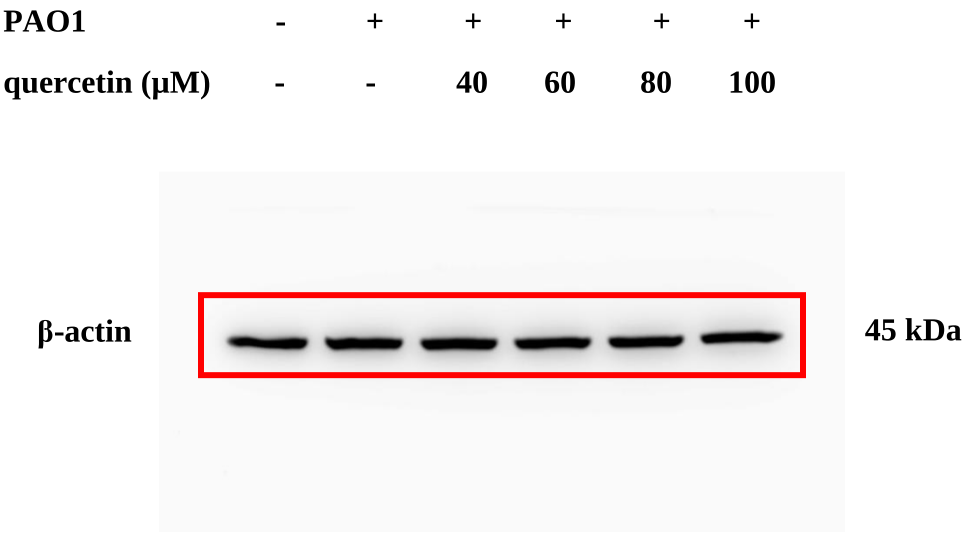

Supplement: S1 Raw images — (PDF) [file pone.0237752.s002.pdf]
