# Supplementary figures and images for: Quercetin negatively regulates IL-1β production in Pseudomonas aeruginosa-infected human macrophages through the inhibition of MAPK/NLRP3 inflammasome pathways
Source: PLoS One. 2020 Aug 20;15(8):e0237752. doi: 10.1371/journal.pone.0237752 (PMC7446918; doi:10.1371/journal.pone.0237752)

**Fig 6A**

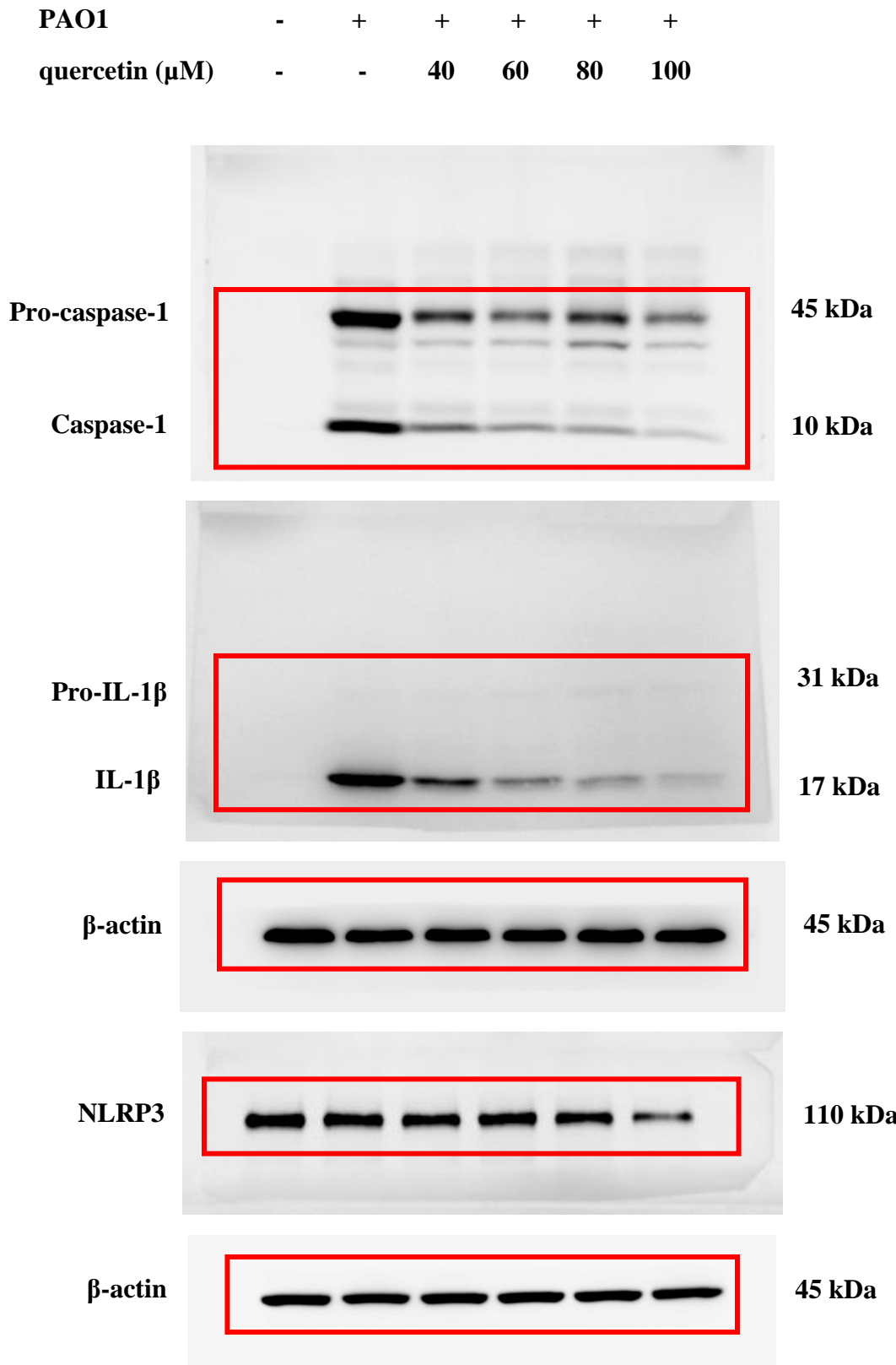

Supplement: S2 Raw images — (PDF) [file pone.0237752.s003.pdf]
